# Supplementary material for: An FGF15/19-TFEB regulatory loop controls hepatic cholesterol and bile acid homeostasis
Source: Nat Commun. 2020 Jul 17;11:3612. doi: 10.1038/s41467-020-17363-6 (PMC7368063; doi:10.1038/s41467-020-17363-6)
Supplement: Supplementary file 1 — Supplementary information [file 41467_2020_17363_MOESM1_ESM.pdf]

An FGF15/19-TFEB regulatory loop controls hepatic cholesterol and bile acid homeostasis

Wang et al.

This file contains:

- Supplemental Figures 1-11
- Supplemental Table 1. Real-time PCR primer sequences

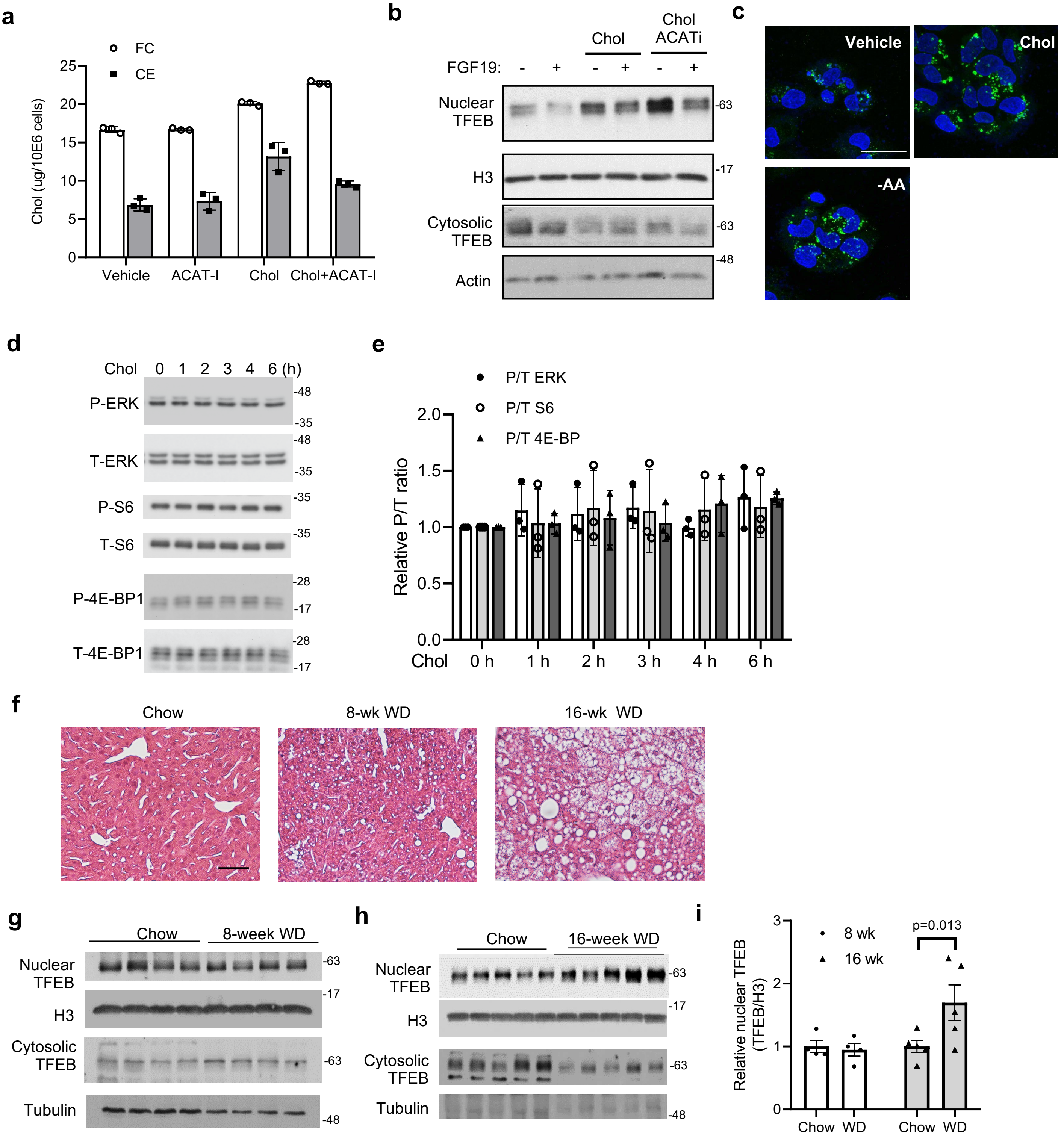

Supplemental Figure 1. Cholesterol promotes TFEB nuclear translocation. a. HepG2 cells were treated with 25 µg/ml cholesterol and 12.5 µg/ml ACAT inhibitor SANDOZ 58-035 (ACATi) for 16 h. FC: free cholesterol. CE: Cholesterol ester. Results were mean ± SD of technical triplicates. **b.** Nuclear and cytosolic TFEB protein. HepG2 cells were treated with 50 ng/ml FGF19 for 30 min followed by 25 µg/ml cholesterol and 12.5 ug/ml ACAT inhibitor SANDOZ 58-035 (ACATi) for 6 h. Representative images of 3 independent experiments with similar results. **c.** HepG2 cells were treated with 25 µg/ml cholesterol for 8 h or cultured in amino acid free EBSS culture medium for 3 h. Immunostaining was performed with anti-Lamp1 antibody. Nuclei were stained with DAPI. Confocal microscope was used to acquire images. Scale bar: 25 um. Images are representative of 4 independent experiments. **d-e.** HepG2 cells were treated with 25 µg/ml cholesterol in time course. Phosphorylated and total ERK, S6 and 4E-BP1 were measured. Mean phosphorylated (P) /total (T) protein ratio of 3 independent experiments were expressed as mean ± SD. **f-i.** Nuclear and cytosolic TFEB in the liver of male C57BL/6J mice fed Western diet for 8 weeks or 16 weeks. Representative images of liver H&E staining of 5 mice/group are shown in "f". Scale bar: 50 um. Relative nuclear TFEB band densitometry of "g" and "h" are shown in "i" as mean ± SEM, n=4 mice/group for "g" and n=5 mice/group for "h". Two-sided Student's t-test was used for "e, i". Source data for a, b, c, d, e, g, h, and i are provided as a Source Data file.

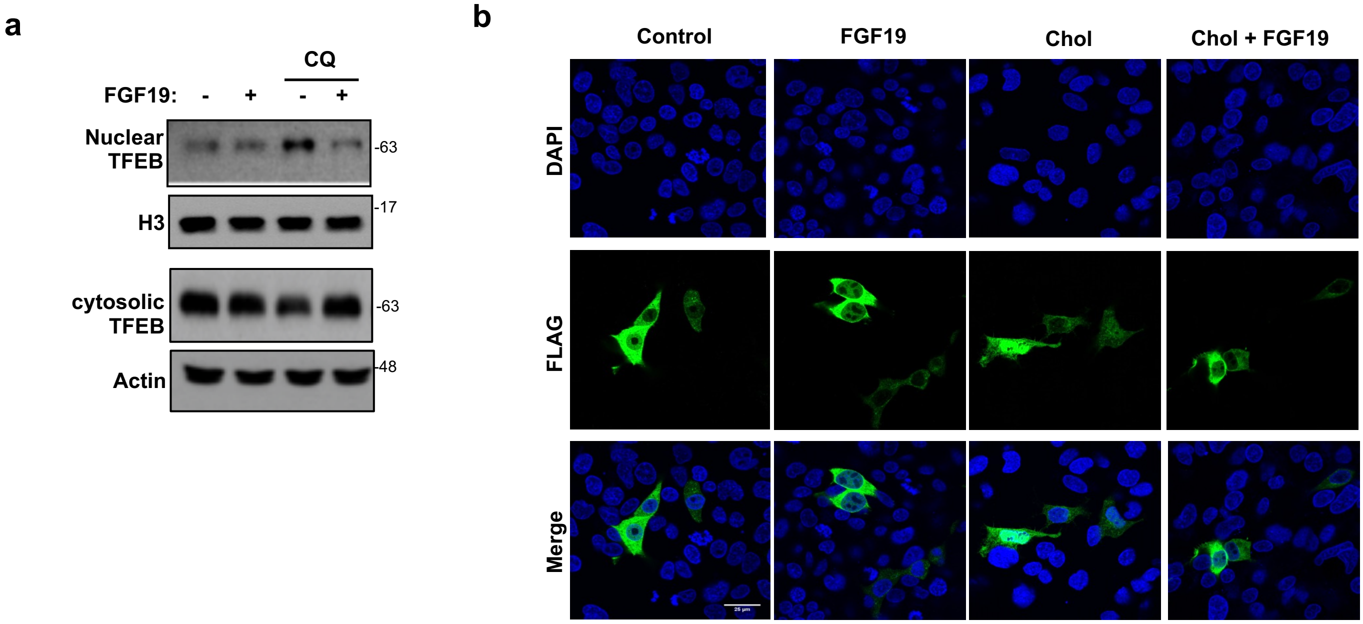

Supplemental Figure 2. FGF19 inhibits TFEB nuclear translocation. a. Nuclear and cytosolic TFEB protein. HepG2 cells were treated with 50 ng/ml FGF19 for 30 min followed by chloroquine (CQ, 50 μM) for 6 h. Images are representative of 2 independent experiments with similar results. **b.** Expanded version of Fig 3f. TFEB-FLAG expression plasmids were transfected in HepG2 cells. Cells were treated with 50 ng/ml FGF19 for 30 min followed by 25 μg/ml cholesterol treatment for 6 h. Immunostaining was performed with anti-FLAG antibody. Nuclei were stained with DAPI. Representative images of 3 independent experiments with similar results are shown. Scale bar: 25 um. Source data for “a” is provided as a Source Data file.

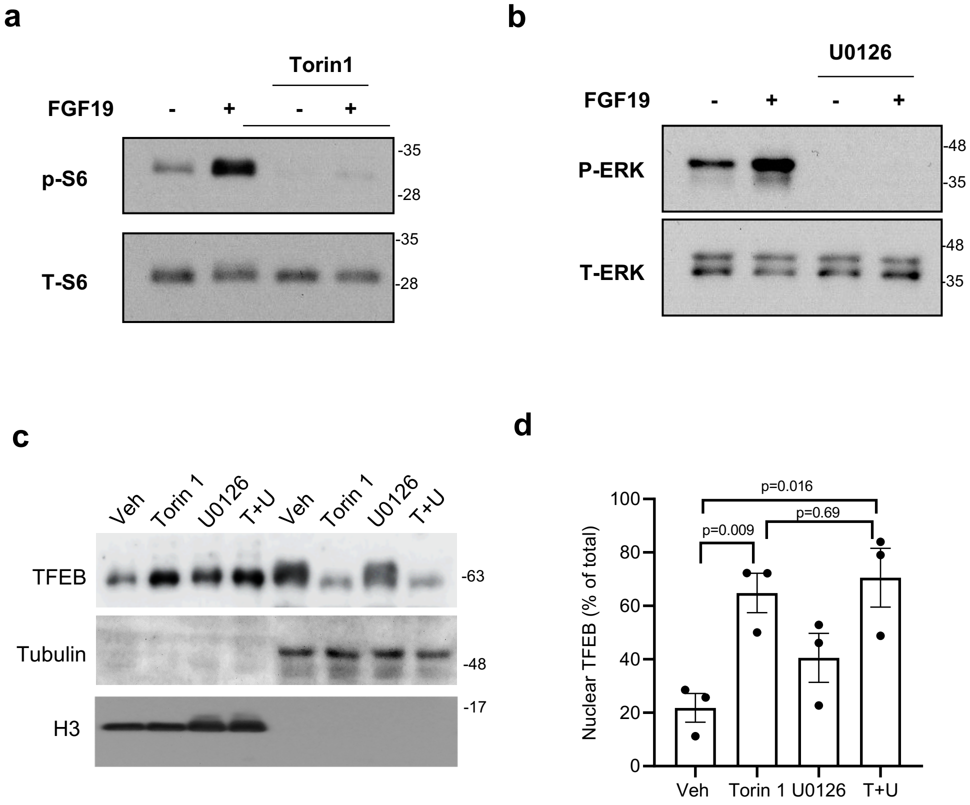

Supplemental Figure 3. Blocking mTOR and ERK signaling increased TFEB nuclear localization in HepG2 cells. a-b. Overnight serum starved HepG2 cells were treated with 250 nM Torin1, 10 μM U0126 and 50 ng/ml FGF19 as indicated for 6 h. Effect on ERK1/2 phosphorylation and mTOR target S6 phosphorylation were measured by Western Blotting. Representative images of 3 independent experiments with similar results. **c-d.** HepG2 cells were treated with 250 nM Torin 1 (T) and/or 10 μM U0126 (U) as indicated for 6 h. **c.** A representative blot of nuclear and cytosolic TFEB protein of 3 independent experiments. H3: histone 3. **d.** Nuclear TFEB as % of total TFEB (nuclear + cytosolic TFEB) was calculated based on densitometry. Mean ± SD of 3 independent experiments was shown. Two-sided Student's t-test was used for "d". Source data for a-d are provided as a Source Data file.

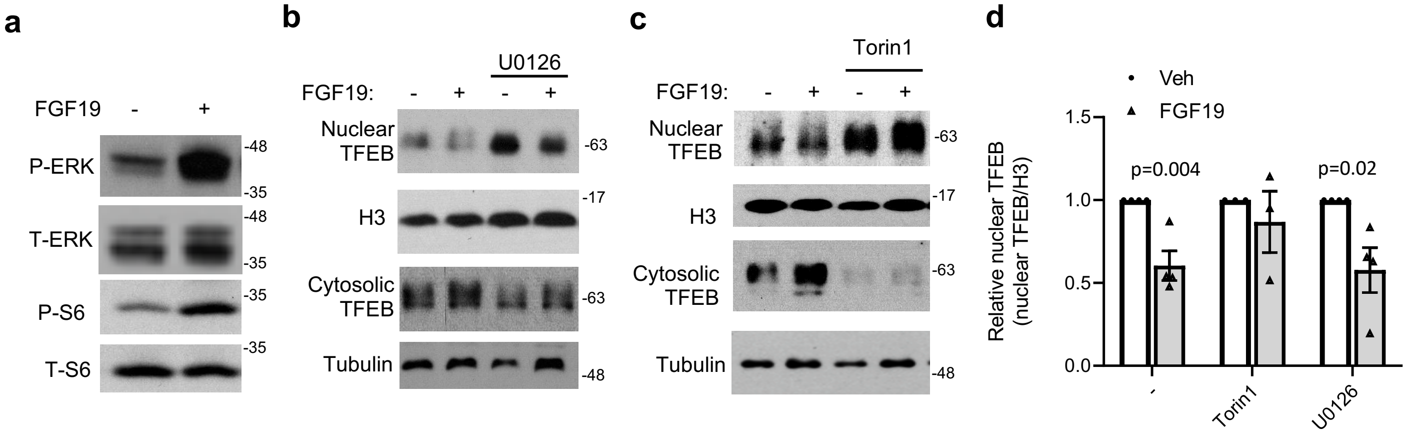

Supplemental Figure 4. Blocking mTOR signaling abolished the inhibitory effects of FGF19 on TFEB nuclear localization in primary human hepatocytes. a. Primary human hepatocytes were treated with 50 ng/ml FGF19 for 6 h. Total and phosphorylated ERK and S6 were measured. Representative images of 4 independent experiments with similar results. **b-d.** Nuclear and cytosolic TFEB abundance in primary human hepatocytes. Cells were treated with vehicle, 250 nM Torin1 or 10 μM U0126 for 30 min followed by 50 ng/ml FGF19 treatment for 6 h. A representative blot is shown in "b" and "c". Average nuclear TFEB abundance of independent preparations of hepatocytes is shown in "d" (n=4 for Veh and U0126, n=3 for Torin1). Value of the non-FGF19 treated group was arbitrarily set as "1". Two-sided Student's t-test was used for "d". Source data for a-d are provided as a Source Data file.

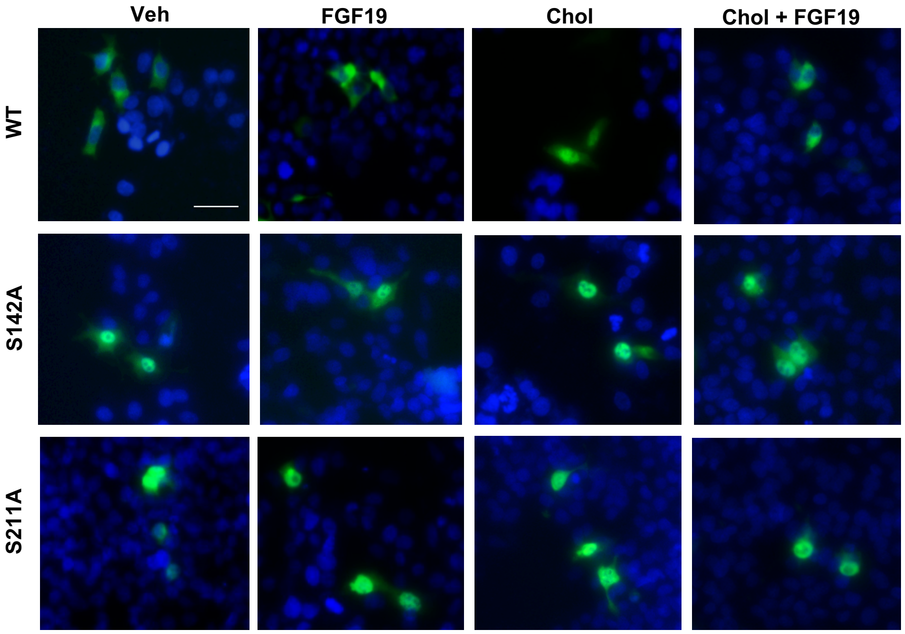

Supplemental Figure 5. FGF19 inhibition of TFEB nuclear localization requires S211 phosphorylation. Representative images of quantitative results of 3 independent experiments shown in Fig 4D. Green. FLAG; blue: DAPI. Scale bar: 50 µm.

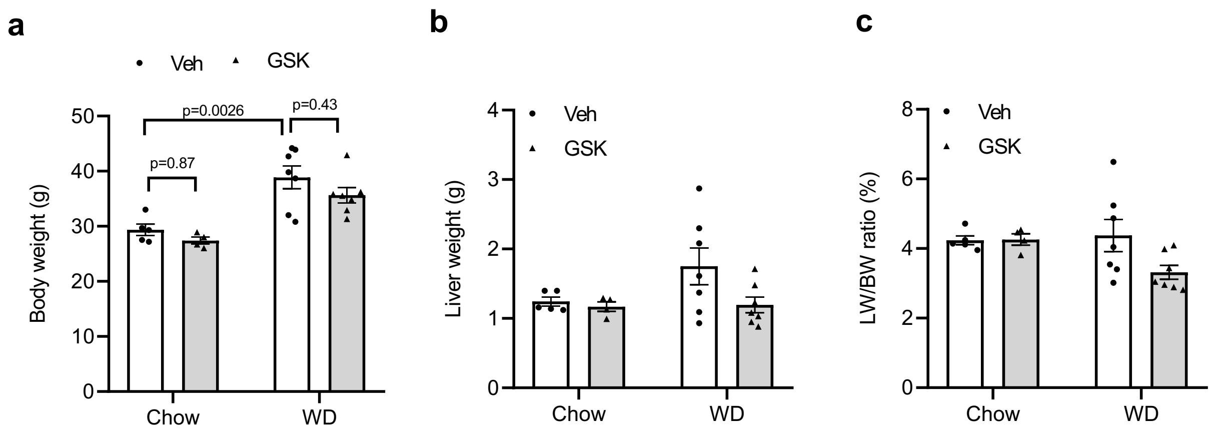

Supplemental Figure 6. Effect of ASBT inhibitor treatment on liver weight to body weight ratio in mice. Male 10-week old C57BL/6J mice were fed chow or WD for 10 weeks, and treated with 2 mg/kg GSK or vehicle (veh) daily via oral gavage for 2 weeks. **a.** Body weight. **b.** Liver weight. **c.** Liver weight (LW) to body weight (BW) ratio. n=5 mice/group for Veh+Chow; n=4 mice/group for GSK+Chow; n=7 mice/group for Veh+WD and GSK+WD. Results were expressed as mean ± SEM. Two-way ANOVA and Tukey post hoc were used for A-C. Source data for a-c are provided as a Source Data file.

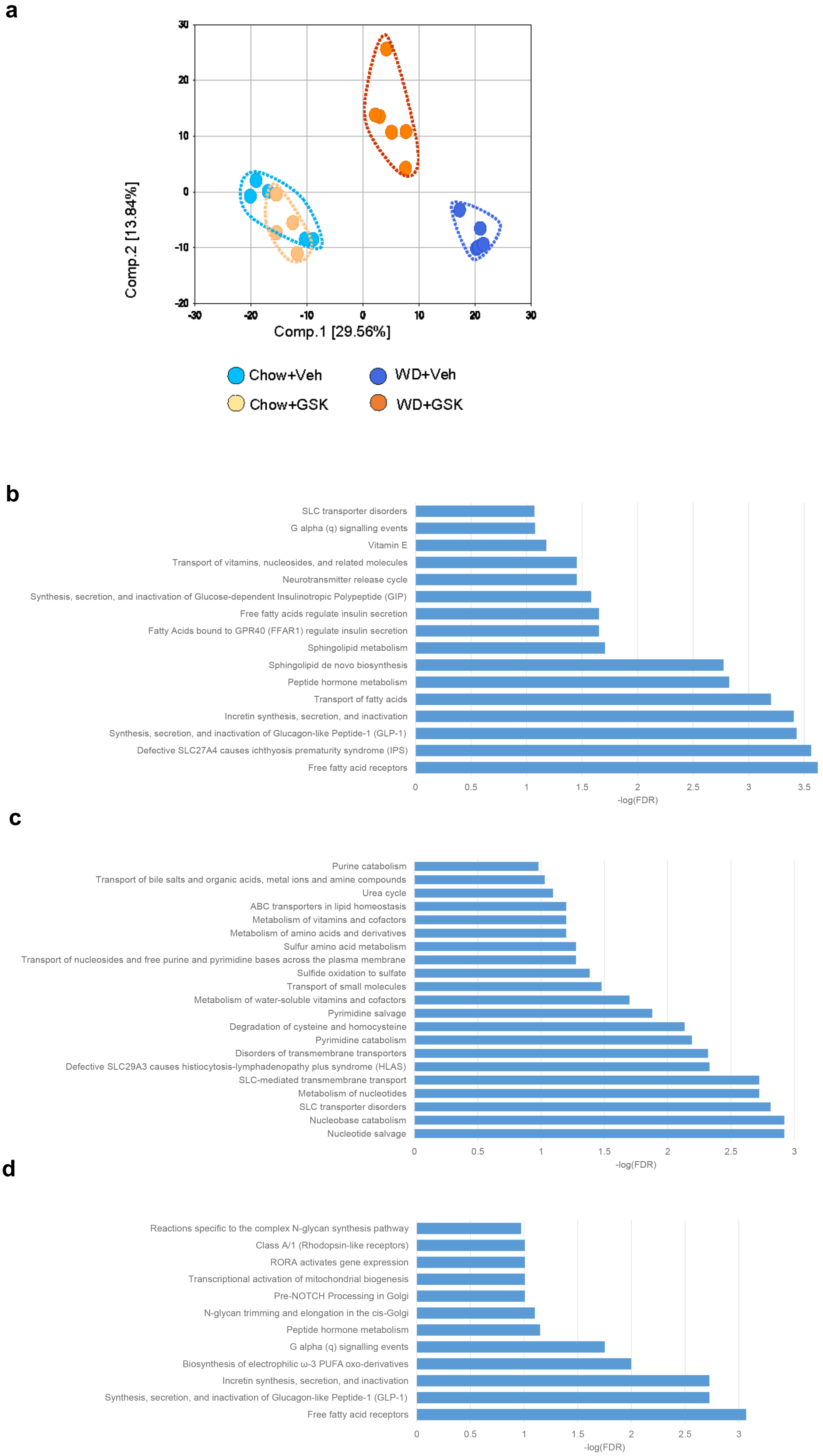

Supplemental Figure 7. Principle components and pathway analysis of hepatic metabolites in GSK-treated mice. Male 10-week old C57BL/6J mice were fed either chow or WD for 10 weeks, and then treated with 2 mg/kg GSK or vehicle (veh) via oral gavage for 2 weeks. Liver metabolites were measured by metabolomics. **a.** Principle component analysis. **b.** Top altered pathways in cluster 1. **c.** Top altered pathways in cluster 2. **d.** Top altered pathways in cluster 3. This analysis is relevant to clustering analysis shown in Fig 7e and is described in the "Methods" section.

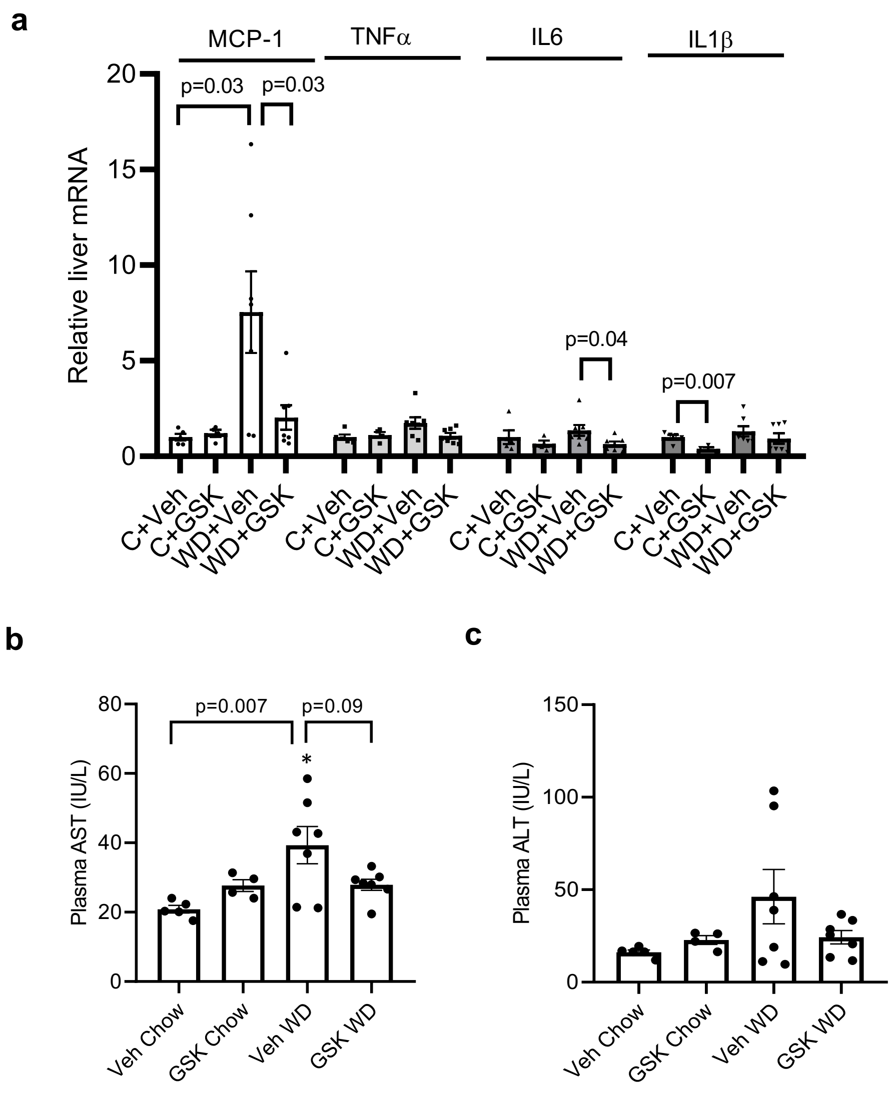

Supplemental Fig 8. Effects of GSK treatment on hepatic cytokine mRNA expression and plasma transaminases in mice. a. Hepatic mRNA expression of monocyte chemoattractant protein 1 (MCP-1), Tumor necrosis factor α (TNFα), interleukin-6 (IL6) and interleukin-1β (IL1β). **b-c.** Plasma aspartate aminotransferase (AST) and alanine aminotransferase (ALT). n=5 mice/group for Veh+Chow; n=4 mice/group for GSK+Chow; n=7 mice/group for Veh+WD and GSK+WD. Results were expressed as mean ± SEM. Two-way ANOVA and Tukey post hoc were used for b. Two-way ANOVA yielded insignificant p values (>0.05) for a and c. Two-sided Student's t-test was used to compare GSK effect in "a" and p values < 0.05 were shown. Source data for a-c are provided as a Source Data file.

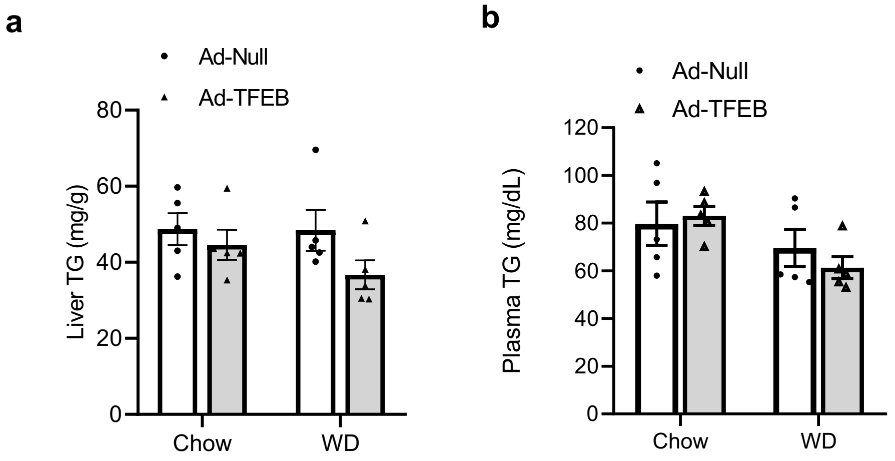

Supplemental Figure 9. Effects of hepatic TFEB overexpression on hepatic and plasma TG in mice. Male 10-week old C57BL/6J mice were injected Ad-Null or Ad-TFEB at a dose of $5X10^8$ pfu/mouse via tail vein. Mice were fed chow diet for one week and then either fed chow or challenged with WD for one additional week. (n=5 mice/group). **a.** Liver TG. **b.** Plasma TG. All results were expressed as mean ± SEM. Two-way ANOVA and Tukey post hoc were used. Source data for a-b are provided as a Source Data file.

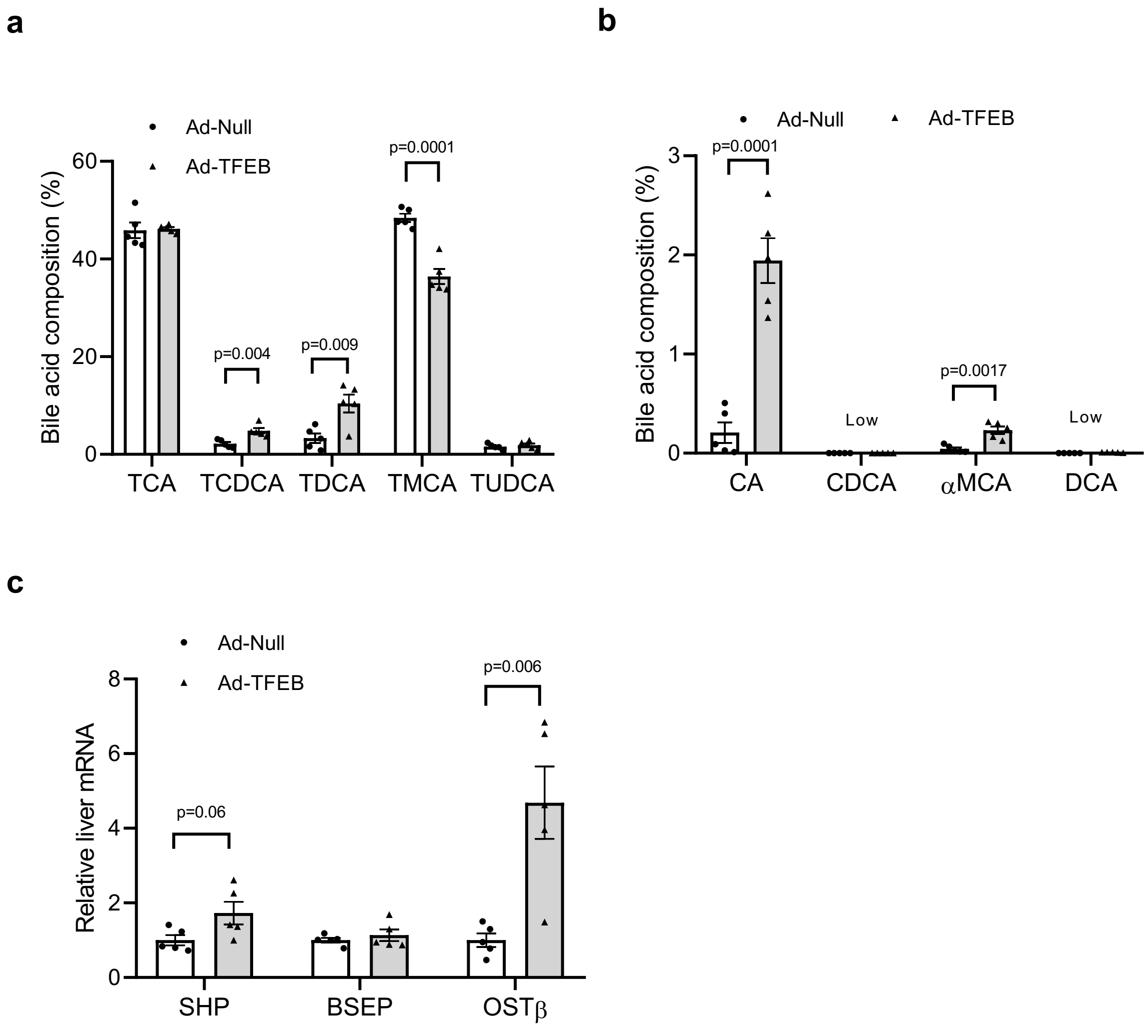

Supplemental Figure 10. Effect of hepatic TFEB overexpression on bile acid composition in chow-fed mice. Male 10-week old C57BL/6J mice were injected Ad-Null or Ad-TFEB at a dose of $5X10^8$ pfu/mouse via tail vein. Mice were fed chow diet for two additional weeks and sacrificed. (n=5 mice/group). **a-b.** Gallbladder bile acid composition was measured with LC-MS method. TMCA is the total of T-αMCA, T-βMCA and T-ωMCA. Low: Less than 0.01% of total bile acid content. **c.** Liver mRNA. All results were expressed as mean ± SEM. Two-sided Student's t-test was used for "a-c". Source data for a-c are provided as a Source Data file.

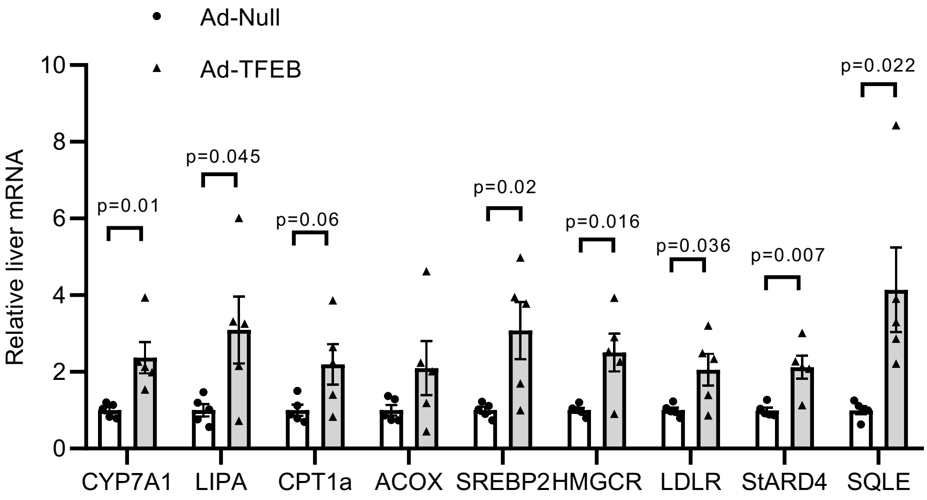

Supplemental Figure 11. Effects of hepatic TFEB overexpression on hepatic gene expression in WD-fed mice. Male 10-week old C57BL/6J mice were injected Ad-Null or Ad-TFEB at a dose of $5X10^8$ pfu/mouse via tail vein. Mice were fed chow diet for one week and then challenged with WD for one additional week. All mRNA expression results were expressed as mean ± SEM. (n=5 mice/group). Two-sided Student's t-test was used. Source data are provided as a Source Data file.

Supplemental Table 1. Real-time PCR primer sequences

m-CYP7A1	F	GGGATTGCTGTGGTAGTGAGC
	R	GGTATGGAATCAACCCGTTGTC
m-CYP8B1	F	CCTCTGGACAAGGGTTTTGTG
	R	GCACCGTGAAGACATCCCC
M-TFEB	F	AAGGTTCGGGAGTATCTGTCTG
	R	GGGTTGGAGCTGATATGTAGCA
m-PGC1α	F	AGACGGATTGCCCTCATTTGA
	R	GGTCTTAACAATGGCAGGGTTT
m-CPT1	F	CTCCGCCTGAGCCATGAAG
	R	CACCAGTGATGATGCCATTCT
m-FGF21	F	CTGCTGGGGGTCTACCAAG
	R	CTGCGCCTACCACTGTTCC
m-FGF15	F	ATGGCGAGAAAGTGGAACGG
	R	CTGACACAGACTGGGATTGCT
m-LIPA	F	TGTTCGTTTTCACCATTGGGA
	R	CGCATGATTATCTCGGTCACA
m-MCP1	F	TTAAAAACCTGGATCGGAACCAA
	R	GCATTAGCTTCAGATTTACGGGT
m-TNFα	F	CCCTCACACTCAGATCATCTTCT
	R	GCTACGACGTGGGCTACAG
m-IL6	F	TAGTCCTTCCTACCCCAATTTCC
	R	TTGGTCCTTAGCCACTCCTTC
m-IL1β	F	GCAACTGTTCCTGAACTCAACT
	R	ATCTTTTGGGGTCCGTCAACT
m-SHP	F	TGGGTCCCAAGGAGTATGC
	R	GCTCCAAGACTTCACACAGTG
m-BSEP	F	ATGTGACCTTCCATTATCCTTCTCG
	R	GCTGTAGTGCTGTGCTCTTCC
m-OSTβ	F	AGAAACATCTCAATCAGGAGCAG
	R	CTGGTGTTTCTTTGTCTTGTGG
m-ACOX1	F	TAACTTCCTCACTCGAAGCCA
	R	AGTTCCATGACCCATCTCTGTC
m-SREBP2	F	GCAGCAACGGGACCATTCT
	R	CCCCATGACTAAGTCCTTCAACT
m-HMGCR	F	AGCTTGCCCGAATTGTATGTG
	R	TCTGTTGTGAACCATGTGACTTC
m-LDLR	F	TGACTCAGACGAACAAGGCTG
	R	ATCTAGGCAATCTCGGTCTCC
m-StARD4	F	AGCCCGTGGTCACAGATTG
	R	GCAACTCGCCACTCATCTTC
m-SQLE	F	ATAAGAAATGCGGGGATGTCAC
	R	ATATCCGAGAAGGCAGCGAAC
h-CYP7A1	F	GAGAAGGCAAACGGGTGAAC
	R	GGATTGGCACCAAATTGCAGA
h-CYP8B1	F	CTTGTTCGGCTACACGAAGGA
	R	GCAGGGAGTAGACAAACCTTG
h-PGC1α	F	TCTGAGTCTGTATGGAGTGACAT
	R	CCAAGTCGTTCACATCTAGTTCA
18S	F	GAGCGAAAGCATTTGCCAAG
	R	GGCATCGTTTATGGTCGGAA
