# Supplementary material for: An FGF15/19-TFEB regulatory loop controls hepatic cholesterol and bile acid homeostasis
Source: Nat Commun. 2020 Jul 17;11:3612. doi: 10.1038/s41467-020-17363-6 (PMC7368063; doi:10.1038/s41467-020-17363-6)
Supplement: Supplementary file 3 — Reporting Summary [file 41467_2020_17363_MOESM3_ESM.pdf]

nature research

Corresponding author(s): Tiangang Li

Last updated by author(s): 6/11/2020

Reporting Summary

Nature Research wishes to improve the reproducibility of the work that we publish. This form provides structure for consistency and transparency in reporting. For further information on Nature Research policies, see Authors & Referees and the Editorial Policy Checklist .

Statistics

For all statistical analyses, confirm that the following items are present in the figure legend, table legend, main text, or Methods section.

n/a	Confirmed	
☐	☒	The exact sample size (*n*) for each experimental group/condition, given as a discrete number and unit of measurement
☐	☒	A statement on whether measurements were taken from distinct samples or whether the same sample was measured repeatedly
☐	☒	The statistical test(s) used AND whether they are one- or two-sided<br>*Only common tests should be described solely by name; describe more complex techniques in the Methods section.*
☒	☐	A description of all covariates tested
☐	☒	A description of any assumptions or corrections, such as tests of normality and adjustment for multiple comparisons
☐	☒	A full description of the statistical parameters including central tendency (e.g. means) or other basic estimates (e.g. regression coefficient) AND variation (e.g. standard deviation) or associated estimates of uncertainty (e.g. confidence intervals)
☐	☒	For null hypothesis testing, the test statistic (e.g. *F*, *t*, *r*) with confidence intervals, effect sizes, degrees of freedom and *P* value noted<br>*Give P values as exact values whenever suitable.*
☒	☐	For Bayesian analysis, information on the choice of priors and Markov chain Monte Carlo settings
☒	☐	For hierarchical and complex designs, identification of the appropriate level for tests and full reporting of outcomes
☐	☒	Estimates of effect sizes (e.g. Cohen's *d*, Pearson's *r*), indicating how they were calculated

Our web collection on statistics for biologists contains articles on many of the points above.

Software and code

Policy information about availability of computer code

Data collection	reactome: https://reactome.org/
Data analysis	GraphPad Prism 6., R v3.5.1, ImageJ, MATLAB R2017a,

For manuscripts utilizing custom algorithms or software that are central to the research but not yet described in published literature, software must be made available to editors/reviewers. We strongly encourage code deposition in a community repository (e.g. GitHub). See the Nature Research guidelines for submitting code & software for further information.

Data

Policy information about availability of data

All manuscripts must include a data availability statement. This statement should provide the following information, where applicable:
- Accession codes, unique identifiers, or web links for publicly available datasets
- A list of figures that have associated raw data
- A description of any restrictions on data availability

The authors declare that data supporting the findings of this study are available within the paper and its supplementary information files. Extended information is available either in the Source Data file or upon reasonable request.

Field-specific reporting

Please select the one below that is the best fit for your research. If you are not sure, read the appropriate sections before making your selection.

☒ Life sciences ☐ Behavioural & social sciences ☐ Ecological, evolutionary & environmental sciences

Life sciences study design

All studies must disclose on these points even when the disclosure is negative.

[illegible]ample size	The sample size was determined based on power analysis (alpha=0.05; beta=0.2) taking into consideration of our existing data. Sample size (n) of each experiment is provided in the corresponding figure captions in the main manuscript and supplementary information files. Sample sizes were chosen based on IACUC approved sample size that are expected to support meaningful conclusions but also minimize the number of animals needed for the study.
Data exclusions	no data exclusion
Replication	Exact number of repeated experiments were stated in Figure legends and statistically analysis was performed when suitable
[illegible]andomization	Mice were ordered from the Jackson Lab. They were assigned to each cage based on how they were divided in the crates. Each cage was randomly chosen to receive treatments. Studies with a single cell line do not involve independent biological samples and do not require randomization.
[illegible]linding	There was no blinding involved in the reported experiments because the objective measurement methods do not introduce bias.

Reporting for specific materials, systems and methods

We require information from authors about some types of materials, experimental systems and methods used in many studies. Here, indicate whether each material, system or method listed is relevant to your study. If you are not sure if a list item applies to your research, read the appropriate section before selecting a response.

Materials & experimental systems

n/a	Involved in the study	
☐	☒	Antibodies
☐	☒	Eukaryotic cell lines
☒	☐	Palaeontology
☐	☒	Animals and other organisms
☒	☐	Human research participants
☒	☐	Clinical data

Methods

n/a	Involved in the study	
☒	☐	ChIP-seq
☒	☐	Flow cytometry
☒	☐	MRI-based neuroimaging

Antibodies

[illegible]ntibodies used	Anti-TFEB antibody (A303-673A) was purchased from Bethyl Laboratoryies, Inc (Montgomery, TX). Actin antibody (ab3280) and alpha-tubulin antibody (ab7291) were purchased from Abcam (Cambridge, MA). FLAG antibody (F1804) was purchased from Sigma-Aldrich (St. Louis, MO). Antibodies against p-ERK1/2 (T202/Y204, #4370), total ERK1/2 (#9102), phospho-S6 ribosomal protein (#2215), total S6 ribosomal protein (#2217), p-4E-BP1 (Thr37/46, #2855), total 4E-BP1 (#9644), and histone 3 (#9717), Normal rabbit IgG (#2729S) were purchased from Cell Signaling Technology (Danvers, MA). Lysosomal-associated membrane protein-1 (Lamp1, 1D4B) was purchased from the Developmental Studies Hybridoma Bank (Iowa City, IA). Alexa Fluor 488-conjugated secondary antibody (A32723) was purchased from Thermo Fisher Scientific, (Grand Island, NY).
[illegible]alidation	TFEB antibody was validated by overexpression and knockdown liver samples. FLAG antibody was validated by cells expressing FLAG-tagged protein. ERK, 4E-BP1 and S6 antibodies were validated by positive control experiments where a chemical inhibitor of ERK and mTOR abolished the phosphorylation of ERK, 4E-BP1 and S6. Lamp-1 antibody was validated by the subcellular localization and changes of Lamp-1 positive puncta in response to cholesterol loading and to amino acid starvation. Actin, Tubulin and histone 3 antibodies were validated by the manufacturer and also confirmed by molecular weight on the SDS-PAGE, and their differential enrichment in nuclear and cytosolic fractions. Normal Rabbit IgG was validated by the manufacturer by expected MW and in a few immunoprecipitation experiments as negative control.

Eukaryotic cell lines

Policy information about cell lines

[illegible]ell line source(s)	HepG2 cells were purchased from ATCC. HEK293A cells were purchased from Invitrogen
[illegible]uthentication	CYP7A1 expression, which is a liver-cell specific gene, was detected in HepG2 cells. No additional authentication procedure was used. HEK293A cells were authenticated by the ability to allow replication defective adenovirus to replicate after infection.
[illegible]ycoplasma contamination	HepG2 cells and HEK293A cells were not tested for mycroplasma.
Commonly misidentified lines (See ICLAC register)	none used

Animals and other organisms

Policy information about studies involving animals; ARRIVE guidelines recommended for reporting animal research

[illegible]aboratory animals	Male C57BL/6J mice were ordered from the Jackson Lab. The sex and age were specified in Figure Legend.
Wild animals	wild animals were not used
[illegible]ield-collected samples	no field-collected samples were used
[illegible]thics oversight	Animal protocols were approved by the Institutional Animal Care and Use Committee at the University of Kansas Medical Center

Note that full information on the approval of the study protocol must also be provided in the manuscript.
