# Supplementary material for: An FGF15/19-TFEB regulatory loop controls hepatic cholesterol and bile acid homeostasis
Source: Nat Commun. 2020 Jul 17;11:3612. doi: 10.1038/s41467-020-17363-6 (PMC7368063; doi:10.1038/s41467-020-17363-6)

Figure 1a

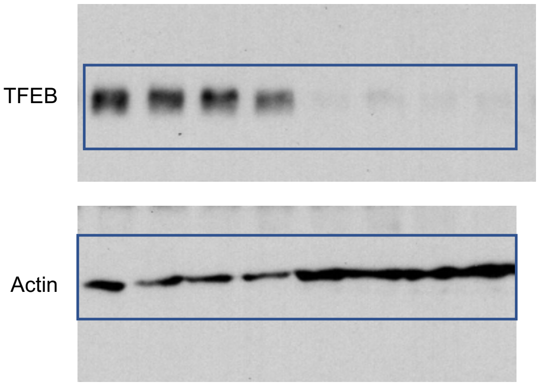

Figure 1c

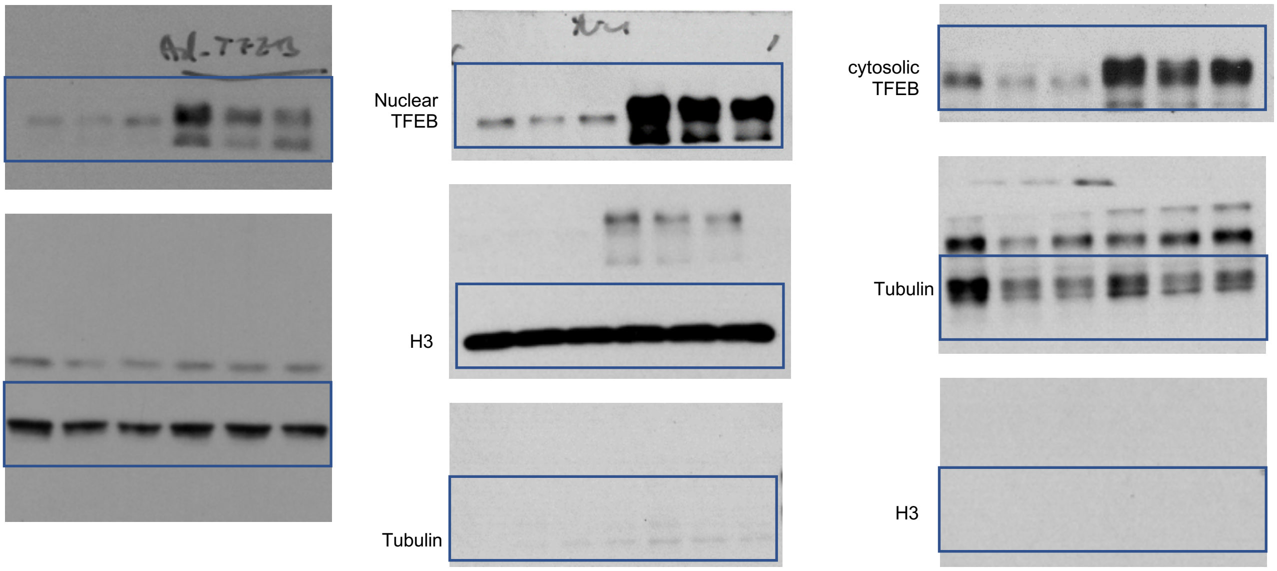

Figure 1f

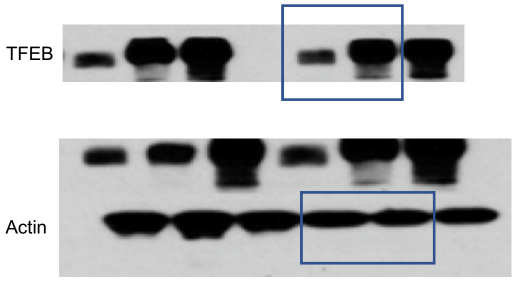

Figure 2b

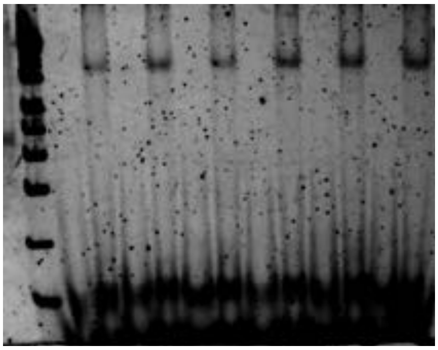

Figure 2c

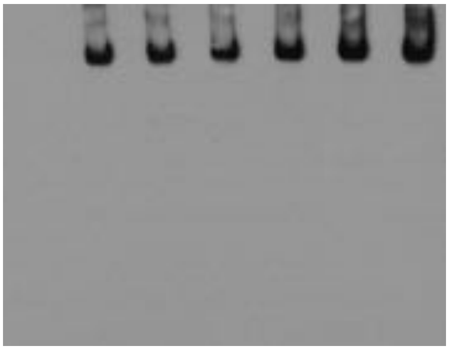

Figure 3b

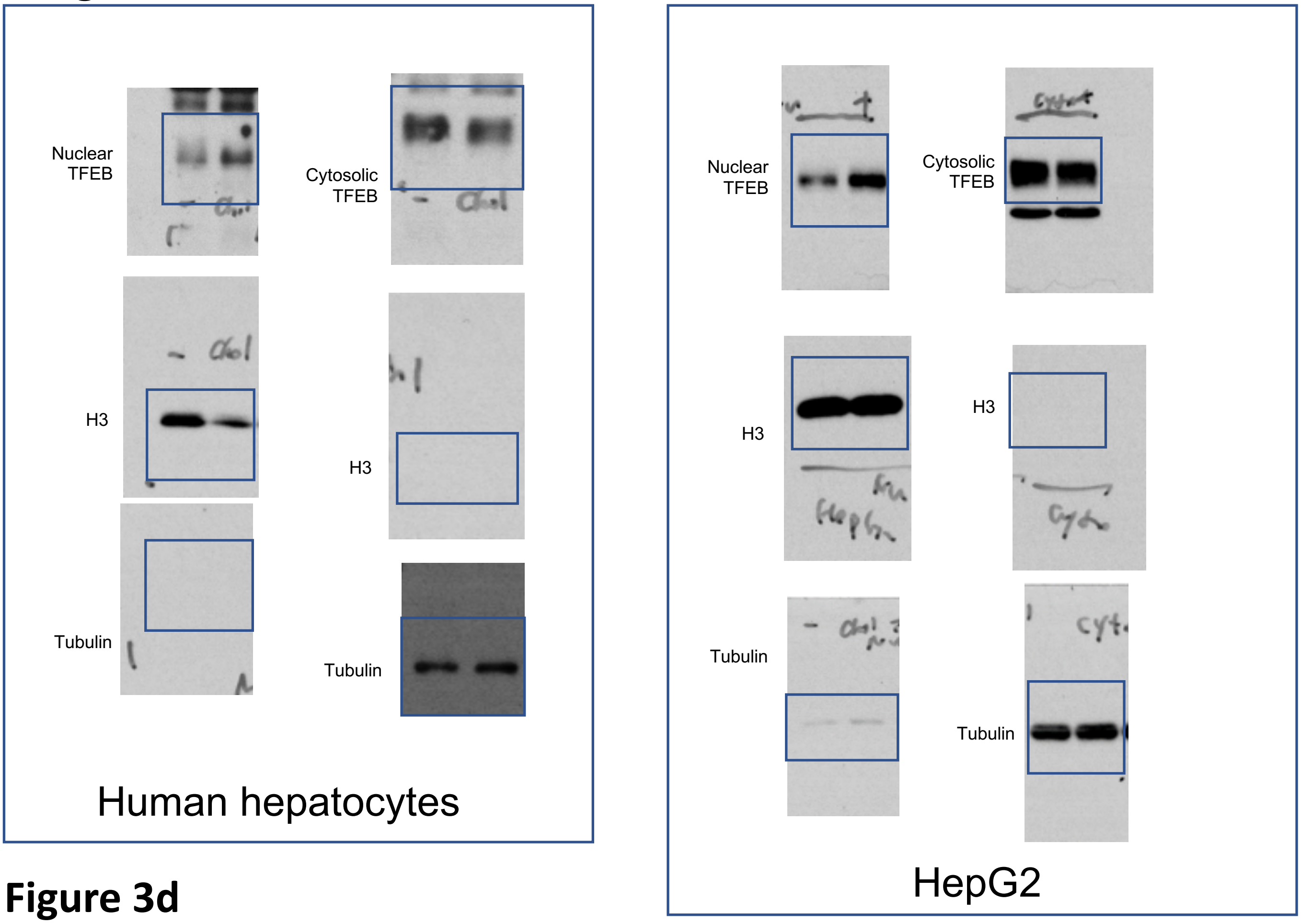

Figure 3d

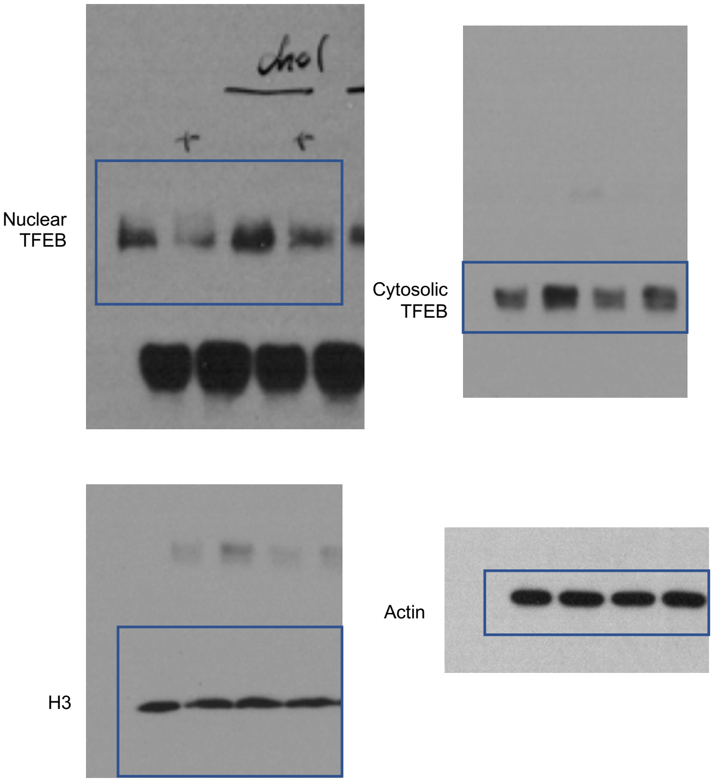

Figure 3e

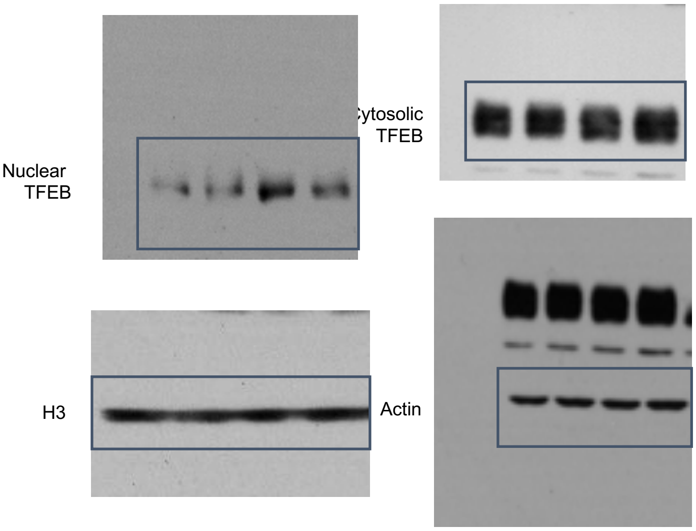

Figure 4a

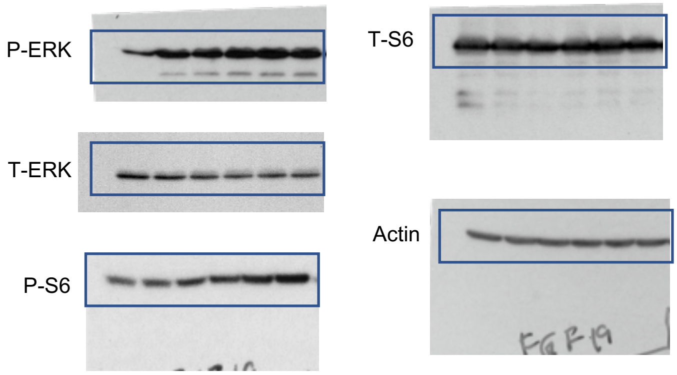

Figure 4b

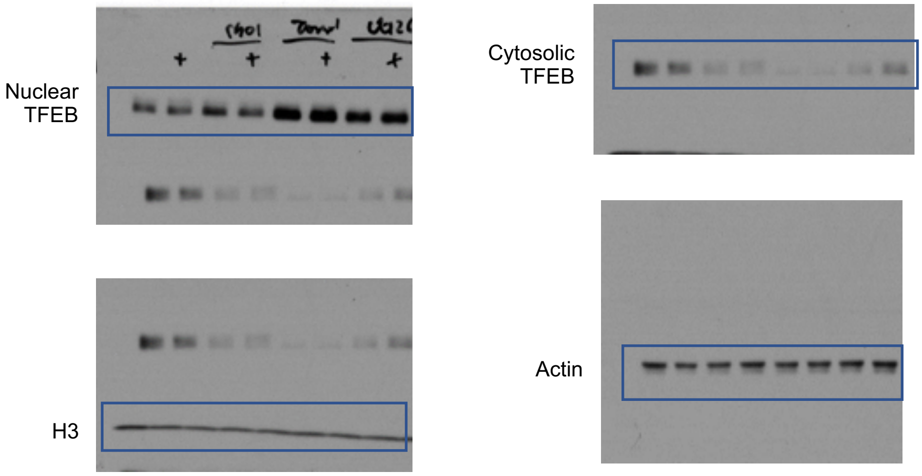

Figure 4e

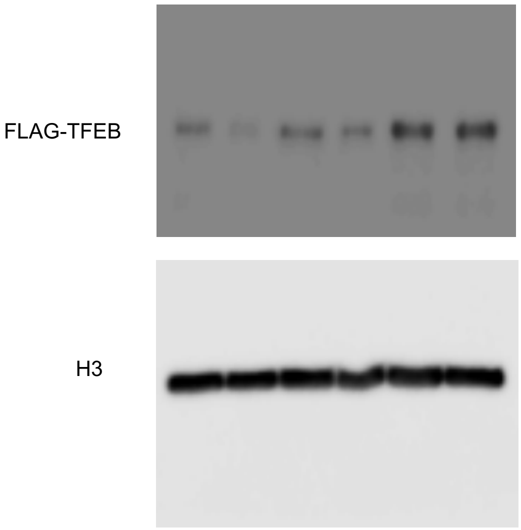

Figure 5a

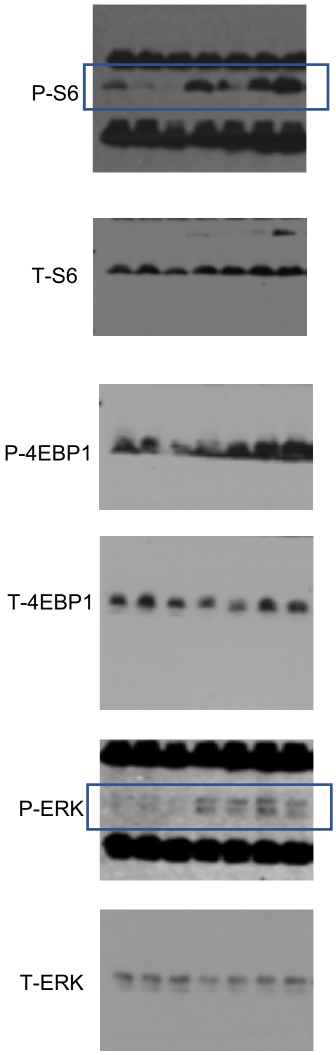

Figure 5c

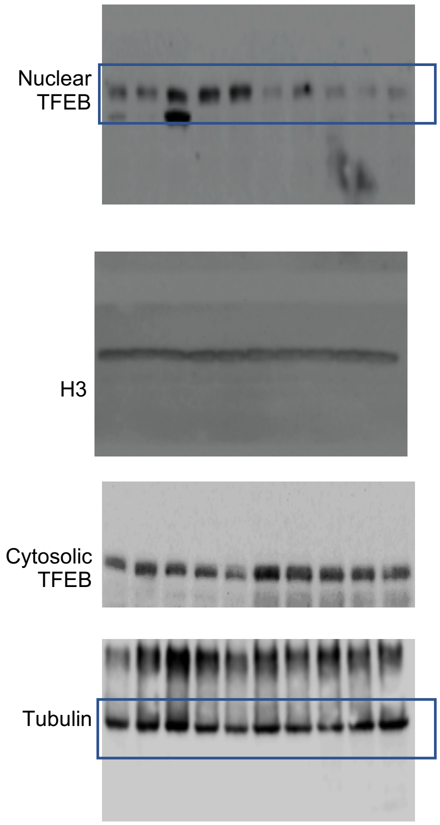

Figure 5e

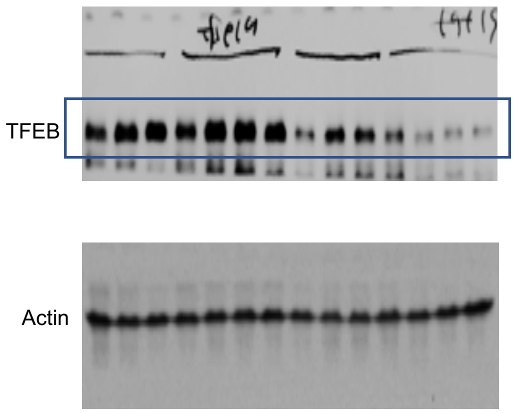

Figure 6d

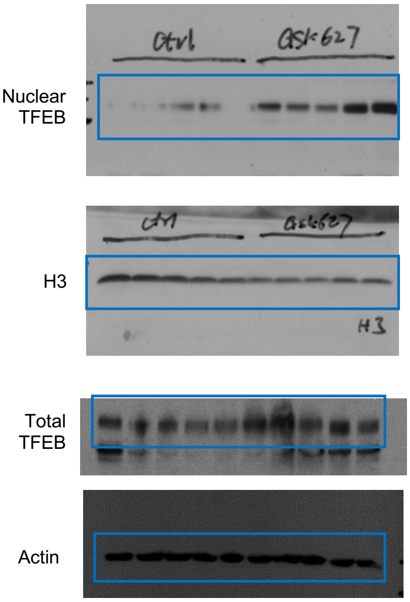

Figure 7a

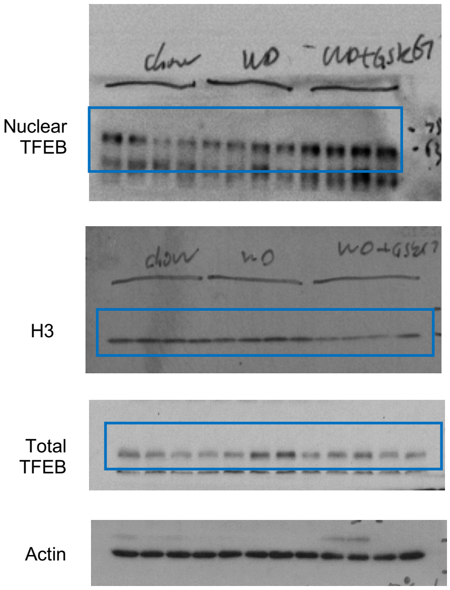

Figure 9a

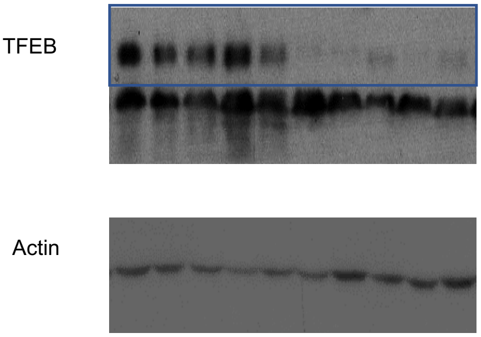

Supplemental Figure 1b

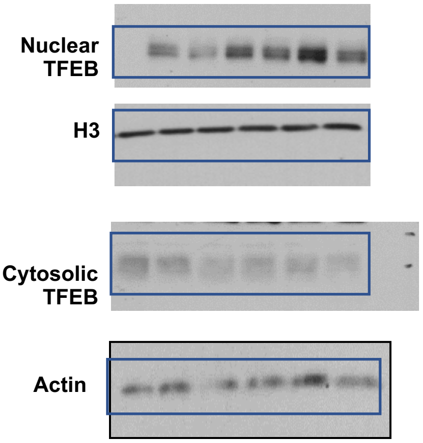

Supplemental Figure 1d

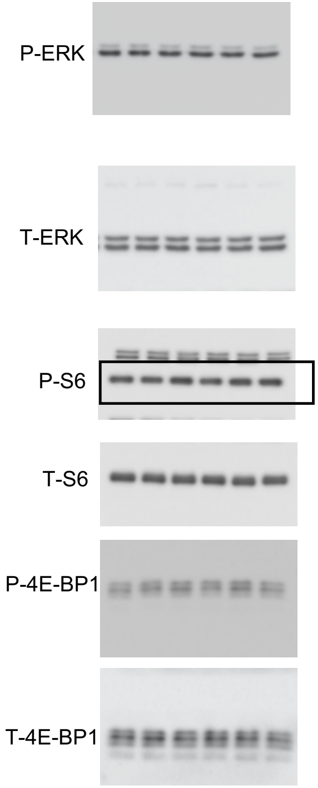

Supplemental Fig 1g

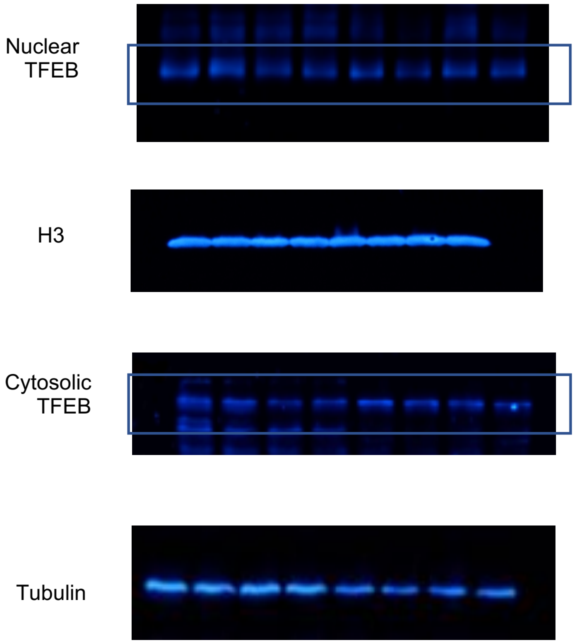

Supplemental Fig 1h

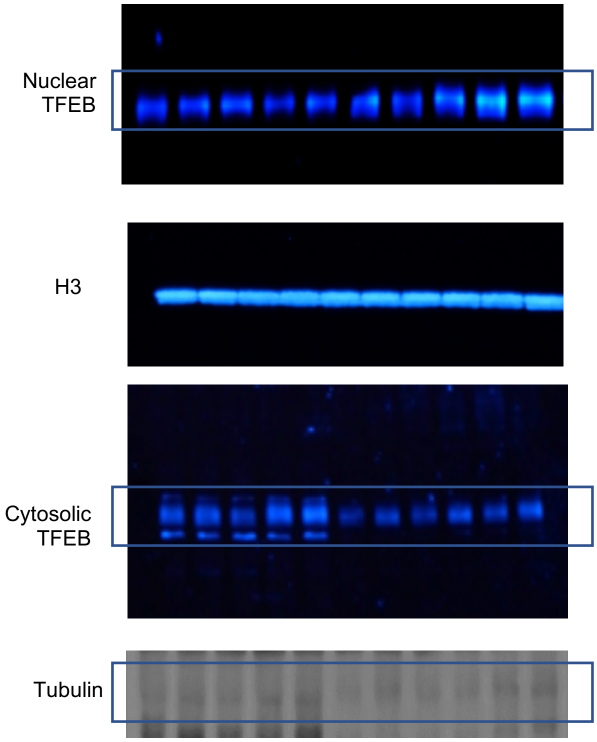

Supplemental Figure 2a

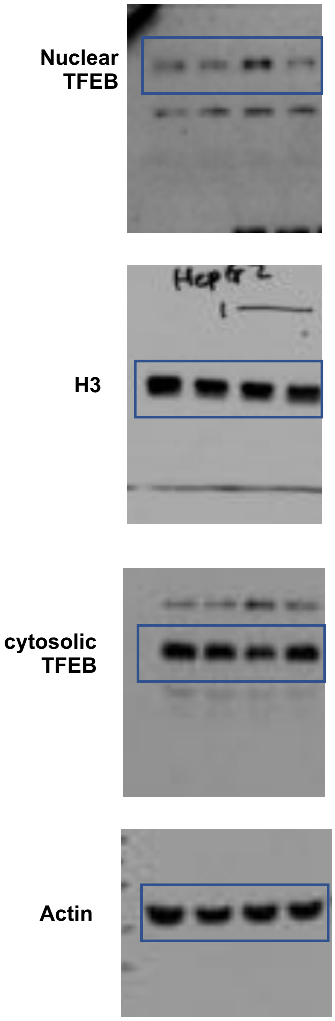

Supplemental Figure 3a

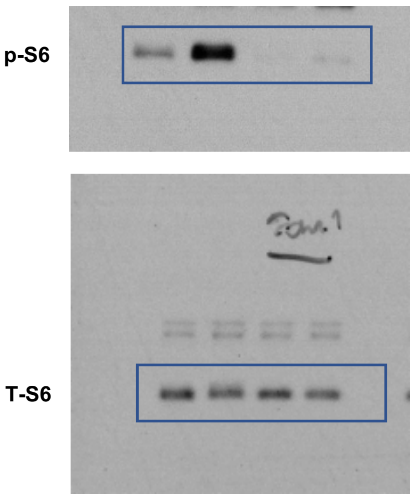

Supplemental Figure 3b

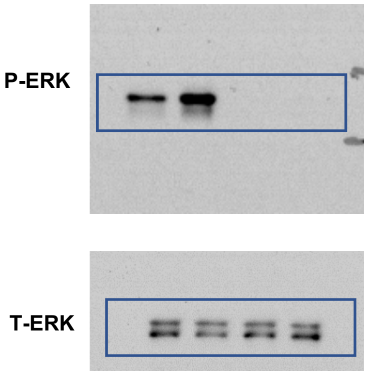

Supplemental Figure 3c

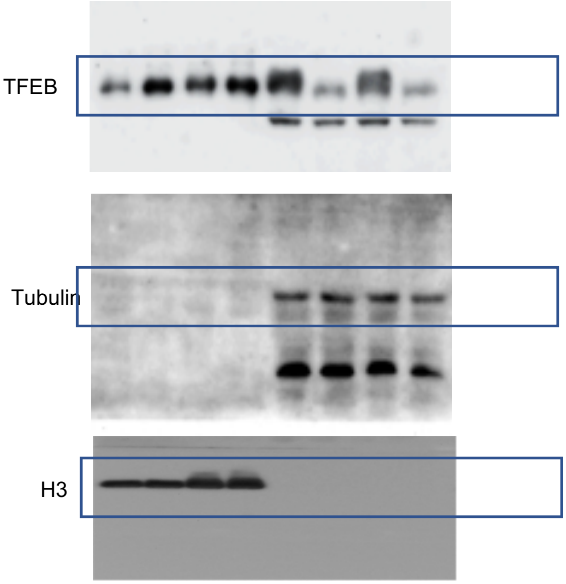

Supplemental Figure 4a

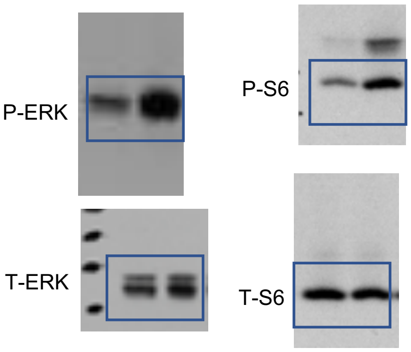

Supplemental Figure 4b

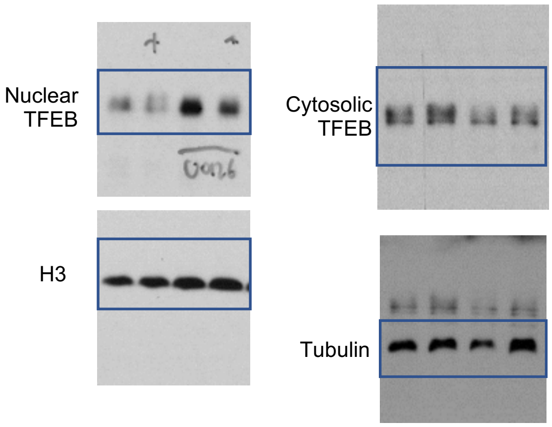

Supplemental Figure 4c

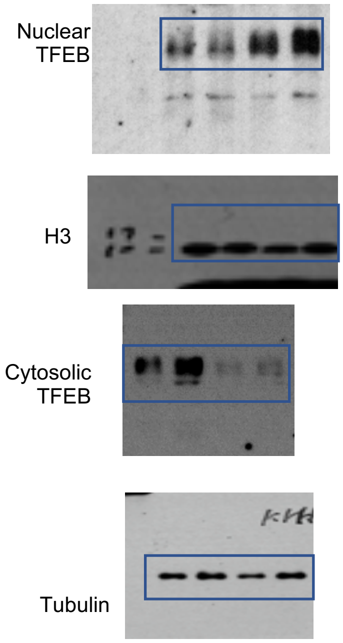

Supplement: Supplementary file 4 — Source Data [file 41467_2020_17363_MOESM4_ESM.zip › Source Data_Wang_et_al/Gel Blots_Wang_et_al.pdf]
